# Urban–Rural Disparities in Knowledge, Use and Perceived Benefits of Nutrition Labels in China: Evidence from 10 Provinces

**DOI:** 10.3390/nu15051171

**Published:** 2023-02-26

**Authors:** Linlin Fan, Zhigang Wang, Yiwen Zhao, Ye Ma

**Affiliations:** 1 Department of Agricultural Economics, Sociology and Education, The Pennsylvania State University, State College, PA 16801, USA; 2 School of Agricultural Economics and Development, Renmin University of China, Beijing 100872, China; 3 Information Center, Ministry of Agriculture and Rural Affairs, Beijing 100125, China

**Keywords:** nutrition labels, urban–rural disparities, Oaxaca–Blinder decomposition

## Abstract

There exist significant gaps in nutritional status between urban and rural populations in China. The previous literature has shown that more knowledge and usage of nutrition labels are instrumental in improving diet quality and health. The aim of the study is to analyze: (1) Are there urban–rural disparities in consumer knowledge, use and perceived benefits of nutrition labels in China; (2) If so, what are the magnitudes of the disparities; (3) What can explain the disparities, and how can the disparities be reduced? The Oaxaca–Blinder (O-B) decomposition is utilized to analyze the predictors of urban–rural disparities in nutrition labels based on a self-conducted study of Chinese individuals. The information from a total of 1635 individuals (aged 11–81 years) across China in 2016 was collected in the survey. We find that rural respondents have less knowledge, lower usage and perceived benefits of nutrition labels than their urban counterparts. Demographics, focus on food safety, frequent shopping locations and income jointly explain 98.9% of the disparity in the knowledge of nutrition labels. Nutrition label knowledge is the predictor which contributes most to urban–rural disparity in label use—accounting for 29.6% of the disparity. Nutrition label knowledge and use are the two biggest predictors of disparities in perceived benefits—accounting for 29.7% and 22.8% of the disparity in perceived benefits, respectively. Our study suggests that policies aiming to improve income and education, as well as raising awareness of food safety in rural areas, are promising in closing the urban–rural disparities in nutrition labels knowledge, use, diet quality and health in China.

## 1. Introduction

As China’s economy has taken off in the past decades, diet-related diseases have also gradually increased. An increasing number of people suffer from various chronic non-communicable diseases because of unhealthy diets [1]. Poor diets are a major contributor to non-communicable diseases that account for over 80% of deaths in China every year [2]. The China State Council Information Office reports that chronic diseases account for 88.5% of all deaths in 2019 [3]. Diet-related health problems, such as obesity, hypertension, type II diabetes and cardiovascular diseases, reduce people’s quality of life and place heavy medical expenditures burdens on both individual families and society [4,5]. The excessive consumption of saturated fat, added sugar, sodium and calories in part lead to sharp increases in those diet-related diseases and existing research shows that nutrition labels about the nutrient contents of foods help consumers make better dietary choices [6,7,8]. For example, nutrition label uses are found to reduce individuals’ daily calorie intake from saturated fat, cholesterol and sodium by 2.1%, 67.6 milligrams and 29.58 milligrams, respectively, while increasing average daily fiber intake by 7.51 g [6]. Consumers who frequently use nutrition labels have better diets and lower risks of diet-related comorbidities [9,10,11,12,13].

China has established mandatory nutrition labeling since 1 January 2013 [14]. The China “Guidance of Nutrition Labelling of Pre-packaged Food” (GB28050-2011) required mandatory and standardized declaration of essential nutrient content, including energy, protein, fat, carbohydrates, sodium and their respective percentages of nutrient reference values for all pre-packaged food. Despite the fact that mandatory nutrition labeling has been in effect for quite a few years, the knowledge and use of nutrition labels are still very low among Chinese consumers, particularly among rural residents [15,16]. Notably, in spite of rapid economic development in China, significant urban–rural disparities persist [17,18]. There are some China-specific institutional barriers that keep rural and urban populations apart [19]. The Hukou system, or household registration system, has contributed to the existence of a dual economy between urban and rural sectors [20]. The Hukou system restricts the mobility of labor into urban areas and operates like an “immigration visa” system but for rural migrants to urban areas. It is very difficult for rural residents to permanently change their Hukou and live in the cities so that they can enjoy equal opportunities of education, employment and healthcare as urban residents. There is a recent loosening of restrictions in smaller cities but for megacities, the Hukou system is still in effect. As a result of the dual economy, inequities persist in the level of spending on and access to education, health and social welfare programs between urban and rural areas. To this date, urban–rural disparities are not only shown in education, income and living conditions but also manifest in residents’ access to medical insurance, quality of health services and the availability of safe and quality foods that comply with nutrition labeling regulations. Rural residents also exhibit lower nutritional status and suffer more from diet-related health problems compared with urban residents [21]. Thus, it is imperative to study the factors that may improve their healthy eating behaviors, such as nutrition labels use in this vulnerable population, which could help reduce urban–rural disparities in health.

The knowledge, use and perceived benefits of nutrition labels are low among both urban and rural residents [15,16,22,23]. Several factors may hamper the use and perceived benefits of nutrition labels among Chinese consumers. For example, the information being too technical or complex is commonly cited as the most important reason why nutrition labels are not frequently used [24,25,26]. A recent systematic review revealed that traffic light schemes are more effective in inducing healthy food consumption compared with the Guideline Daily Amount and front-of-package nutrition and health claims [13]. Other factors, such as gender, age, household size, health status, shopping frequency, nutrition knowledge, diet-health concern and income, are found to be significant predictors of nutrition labels usage and knowledge [6,22,23,24,27,28].

Although extensive research has investigated the determinants of nutrition labels’ knowledge, usage and benefits, our study is the first to examine the inequities in the knowledge, use and perceived benefits of nutrition labels between urban and rural consumers in China. Existing studies mostly focus on assessing the use and understanding of nutrition labels among *urban* residents in China. Moreover, data samples usually come from one city or two and thus have limited information on other places in China. In contrast, we conducted a large-scale survey to collect data from both urban and rural areas in 10 provinces across China. This paper aims to answer three questions: (1) Are there urban–rural disparities in consumer knowledge, use and perceived benefits of nutrition labels; (2) If so, what are the magnitudes of the disparities; (3) What can explain the urban–rural disparities in nutrition label knowledge, usage and perceived benefits, and how can the disparities be reduced?

## 2. Data

### 2.1. Study Sample

The data used in this paper came from a survey of urban and rural consumers in Hebei, Jiangsu, Guangdong, Sichuan, Guizhou, Guangxi, Inner Mongolia, Shanxi, Hunan and Heilongjiang conducted by the authors in October and November of 2016. The Renmin University of China Institutional Research Board (IRB) committee approved the study, and parental consent was obtained to survey teenage respondents. Stratified sampling was used, and several provinces were selected from each geographic region of China. Then, one prefecture was selected from each province, and both urban and rural areas in that prefecture were sampled. More than 200 questionnaires were distributed in each prefecture. Individuals aged 11 to 81 years were selected. Because the national urbanization rate is around 60%, we deliberately conducted about 60% of the surveys in urban areas and the remaining 40% in rural areas. Due to missing values in key explanatory and dependent variables, 481 respondents were dropped from the analysis. Another 54 observations were removed because of outliers. As a result, 1635, or 75.3% of all questionnaires, were retained and used in this study. Among the remaining respondents, 696 (42.57%) of them lived in rural areas, and 939 (57.43%) lived in urban areas, which is consistent with the national urbanization rate of 57.35% in 2016.

### 2.2. Outcome Variables

There were three outcome variables in this study—consumers’ knowledge, use and perceived benefits of nutrition labels (Table 1). Three questions were asked, and the corresponding response options were on a 5-point Likert scale. Specifically, the questions were (1) knowledge: “How well do you know about nutrition labels?” with the response options as “1 = do not understand, 2 = understand slightly, 3 = understand moderately, 4 = generally understand, 5 = totally understand”; (2) use: “How often do you use nutrition labels when shopping for food?” with the options as “1 = Never, 2 = Rarely, 3 = Sometimes, 4 = Often, 5 = Always”; and (3) perceived benefits: “How much do you think you have benefited from nutrition labels?” with the options as “1 = no benefits, 2 = benefit slightly, 3 = benefit moderately, 4 = benefit a lot, 5 = benefit very much”.

### 2.3. Explanatory Variables

Motivated by the literature on nutrition labels, the explanatory variables included demographics (the age, BMI and health status of the respondent, whether the respondent graduated from high school and whether the respondent has children or seniors at home), the level of attention paid to food safety issues (never = 1, rarely = 2, sometimes = 3, often = 4 and always = 5) and frequent shopping locations, income and province indicators. Rural residents were defined as those who lived in rural areas for more than six months in a year or otherwise were urban residents. Importantly, gender, marital status and household size were not included in the analysis because these variables were insignificant predictors of the nutrition label knowledge, use and perceived benefits in the regressions. The detailed definition of each variable is also shown in Table 1.

## 3. Methods

Ordinary Least Squares (OLS) regressions were separately performed on the knowledge, use and perceived benefits of nutrition labels among urban and rural respondents to investigate how the significant predictors might differ by region. Next, the Oaxaca–Blinder (O-B) decomposition was used to analyze what explains the disparities in nutrition labels knowledge, use and perceived benefits between urban and rural residents. A *p*-value < 0.1 was deemed statistically significant.

The Oaxaca–Blinder (O-B) decomposition has become a widespread method for decomposing the difference in an outcome between two groups in various research fields [29,30,31]. The popular tool divides the gap between two groups into two parts, which are named as “explained” and “unexplained”. The “explained” part attributes the difference in the gap to the mean of observable predictors between two groups, whereas the different effects of unobservable predictors account for the difference in the gap in the “unexplained” part [32,33].

The objective of this paper is to decompose the gaps in the knowledge, use and perceived benefit level of nutrition labels between urban and rural respondents. The common assumptions about the urban–rural disparities in nutrition labels indicate that the disparities will substantially diminish if society can effectively reduce the disparities in socioeconomic status (SES), such as income and education, and food retail environment between urban and rural residents. The O-B method is particularly helpful when one wants to test how much the urban–rural disparity could still remain in the hypothetical situation where policies successfully improved the above-mentioned predictors for the rural residents such that the mean levels of those indicators became at par with the mean levels for urban residents [30].

Specifically, the disparity in the knowledge of nutrition labels was used as an example to illustrate how the disparity is decomposed based on the O-B decomposition methodology. Initially, assuming that the knowledge level model is linear and includes a set of observable and unobservable predictors, two linear models showing the mean knowledge level for the urban and rural groups are formulated as follows,

(1) $$Knowledge_{u}=X_{u}^{\prime }\beta_{u}+\epsilon_{u}$$

(2) $$Knowledge_{r}=X_{r}^{\prime }\beta_{r}+\epsilon_{r}$$

where *Knowledge* represents the mean knowledge level of nutrition labels, *X* is a vector that consists of predictors and a constant term, β is the corresponding coefficient vector, and *ϵ* is the random error term. The subscript *u* represents the urban group, while subscript *r* represents the rural group. The expected value of the random error term, *ϵ*, is assumed to be zero following the assumption in the linear model.

Then, the means of knowledge level for urban and rural groups can be denoted as below:

(3) $$E\left(Knowledge_{u}\right)=E{\left(X_{u}\right)}^{\prime }\beta_{u}$$

(4) $$E\left(Knowledge_{r}\right)=E{\left(X_{r}\right)}^{\prime }\beta_{r}$$

According to Equations (3) and (4), the difference in mean knowledge level between urban and rural groups can be calculated as follows:

(5) $$R=E{\left(X_{u}\right)}^{\prime }\beta_{u}-E{\left(X_{r}\right)}^{\prime }\beta_{r}$$

Next, overall knowledge difference can be separated into two components based on the O-B decomposition,

(6) $$R=\left[E{\left(X_{u}\right)}^{\prime }-E{\left(X_{r}\right)}^{\prime }\right]\beta^{*}+\left[E{\left(X_{u}\right)}^{\prime }\left(\beta_{u}-\beta^{*}\right)+E{\left(X_{r}\right)}^{\prime }\left(\beta^{*}-\beta_{r}\right)\right]$$

where the first term in Equation (6) is the “explained” part, indicating the difference in the knowledge level between the two groups due to the predictors. The second term represents the “unexplained” part, which contributes to the disparity of the outcome to the difference in the coefficient estimation. This part is also crucial since it contains all potential variables that affect the disparity but are not included in the predictors.

In this paper, the coefficients from pooled regressions combining two groups were used to represent the value of $\beta^{*}$, in order to avoid base group bias and follow common practice in the literature [34,35,36]. Three O-B models are performed to decompose the disparities in knowledge, use and perceived benefits of nutrition labels between rural and urban groups. Specifically, in the first O-B model, the difference in the knowledge between rural and urban groups is decomposed into “explained” and “unexplained” portions. In the second model, the disparity in use between rural and urban groups is analyzed using knowledge as an independent variable. Lastly, both knowledge and use are treated as independent variables in the O-B model, where the differences in perceived benefits between rural and urban consumers are studied.

## 4. Results

### 4.1. Descriptive Statistics

Table 2 summarizes the knowledge, use and perceived benefits of nutrition labels among rural and urban respondents. Results show that rural respondents have less knowledge of nutrition labels—47.55% of rural respondents vs. 38.12% of urban counterparts do not understand nutrition labels. Rural respondents also have lower usage of nutrition labels (rural: 37.35% vs. urban: 22.46%). Lastly, rural respondents also perceive lower benefits from nutrition labels (35.92% of rural vs. 27.05% of urban respondents perceive no benefits or only benefit slightly from nutrition labels). However, a small percentage of both rural and urban respondents (3.62% vs. 3.59%) report benefiting very much from nutrition labels. Similarly, only 1.29% of urban respondents and 2.24% of rural respondents totally understand nutrition labels. Small percentages of rural and urban respondents (7.76% vs. 7.77%) always use nutrition labels when buying food (Table 2).

As can be seen from Table 3, the data in the second and third columns are sample means by urban and rural groups, and rural respondents are more socially and economically disadvantaged. Specifically, the average age of rural respondents is higher than that of urban counterparts (40.58 vs. 31.82, *p*-value < 0.001); rural respondents have higher BMI (23.11 vs. 22.31, *p*-value = 0.001); and fewer rural respondents have at least a high school education than their urban counterparts (51.7% vs. 88.8%, *p*-value < 0.001). Urban residents have better health status, such that 53.7% and 16.6% of them rate their health as good or excellent compared with 42.1% and 17.8% for rural residents (*p*-value < 0.001 and *p*-value = 0.524, respectively). More rural respondents have children and seniors at home (children: 56.5% vs. 51.7%, *p*-value = 0.054; seniors: 47.7% vs. 34.7%, *p*-value < 0.001). Fewer rural respondents often or always pay attention to food safety issues than rural groups (52.8% vs. 59.5%), and rural respondents are less likely to buy food online (21.1% vs. 40.5%, *p*-value < 0.001) or from large supermarkets (60.3% vs. 86.9%, *p*-value < 0.001) than urban residents. Rural respondents have lower annual income than their urban counterparts (4.272 vs. 7.029, *p*-value < 0.001).

### 4.2. OLS Regression Results

Columns (5) and (6) in Table 3 show the OLS regression results on knowledge of nutrition labels against various explanatory variables. Rural respondents who have seniors at home have lower knowledge of nutrition labels. The more often rural respondents pay attention to food safety, the more knowledge of nutrition labels they have. The rural respondents who frequently shop in large supermarkets or online also have more knowledge of nutrition labels. In comparison, rural residents who mostly shop at corner stores report having lower knowledge of nutrition labels (coeff. = −0.138, *p*-value < 0.1). Income is not a significant predictor of nutrition label knowledge among rural respondents. For urban respondents, similarly, the more attention paid to food safety, the more knowledgeable of nutrition labels urban residents are. A higher income also predicts better knowledge of nutrition labels among urban respondents (coeff. = 0.008, *p*-value < 0.1).

Columns (7) and (8) present the OLS regression results on the use of nutrition labels among rural and urban respondents, respectively. More knowledge of nutrition labels is associated with more frequent use of nutrition labels among both rural and urban respondents. The level of attention paid to food safety is also positively associated with the use of nutrition labels for both rural and urban respondents. Rural respondents who frequently shop in large supermarkets use nutrition labels more often (coeff. = 0.264, *p*-value < 0.01), while urban respondents who frequently shop in farmers’ markets use nutrition labels more often (coeff. = 0.100, *p*-value < 0.1). In contrast, urban respondents who mostly shop in corner stores use nutrition labels less often (coeff. = −0.196, *p*-value < 0.01). Similar to the knowledge of nutrition labels, income is a significant and positive predictor of nutrition label use among urban respondents but not in rural respondents.

Lastly, the predictors of perceived benefits from nutrition labels are analyzed among rural and urban respondents (columns (9) and (10) in Table 3). Knowledge and frequent use of nutrition labels are positively associated with perceived benefits from nutrition labels in both rural and urban residents. Age and having a high school education or above are negatively associated with perceived benefits from nutrition labels among rural residents but not among urban respondents. In contrast to the positive association between the level of attention to food safety and knowledge and use of nutrition labels, only urban residents who always pay attention to food safety perceive significantly more benefits from nutrition labels compared with those who never pay attention to food safety (coeff. = 0.444, *p*-value < 0.1). Similarly, although various frequent shopping locations are significant predictors of knowledge and use of nutrition labels among rural respondents, they are not significantly associated with perceived benefits among rural residents after controlling for nutrition label knowledge and usage in the regression. Interestingly, income is negatively associated with perceived benefits from nutrition labels among urban residents (coeff. = −0.009, *p*-value < 0.05).

### 4.3. O-B Decomposition Results

Table 4 shows the O-B decomposition results for the urban–rural disparities in knowledge, use and perceived benefits of nutrition labels. Columns (2) and (3) present the decomposition results for the difference in knowledge between urban and rural respondents. The disparity in the knowledge of nutrition labels between urban and rural groups is 0.183, indicating that urban residents have higher nutrition label knowledge compared with rural groups (also shown in Table 3). A total of 92.84% (*p*-value < 0.01) of the overall disparity in knowledge is explained, which implies that almost all possible predictors are included in the O-B model to explain the disparity. The set of indicator variables denoting focus on food safety together have the largest contribution, accounting for 34.45% of the disparity (*p*-value < 0.01). Specifically, the differences in whether the respondent never (17.27%, *p*-value < 0.01) and often (7.55%, *p*-value < 0.01) focuses on food safety between urban and rural groups contribute most to the disparities among all variables of focus on food safety. As shown in Table 3, urban residents are more likely to pay attention to food safety compared with rural residents. Therefore, if rural residents increased the frequency of focusing on food safety issues, the disparity in nutrition label knowledge would have shrunk. The second largest contributor to the disparity is demographics, accounting for 0.056 out of 0.183 (30.77%, *p*-value < 0.01) in overall disparity between urban and rural respondents. In other words, the average knowledge of nutrition labels in rural residents will raise by 0.056 if they have identical demographic characteristics as the urban residents. The frequent shopping location indicator variables are the third-largest contributor to the disparity, explaining 23.04% of the overall disparity in knowledge between rural and urban residents (*p*-value < 0.05). Lastly, income explains 10.63% of the disparity (*p*-value < 0.05). So, if rural respondents had the same annual income as urban counterparts, 10.6% of the disparity in knowledge of nutrition labels between these two groups would have disappeared.

The second O-B model in Table 4 (Columns (4) and (5)) is to decompose the differences in the use of nutrition labels between rural and urban residents. Table 4 shows that the overall use disparity between these two groups is 0.315, and 66.0% (*p*-value < 0.01) of the overall disparity is explained by predictors contained in the model. This result also suggests that urban residents are more likely to use nutrition labels compared with their rural counterparts (also shown in Table 3). Particularly, we use the knowledge of nutrition labels as an explanatory variable in this O-B model. The knowledge variable has the largest contribution to the overall disparity in the use of nutrition labels among all explanatory variables. Around 30% (*p*-value < 0.01) of the overall disparity in the use of nutrition labels is explained by differences in knowledge. The difference in demographic characteristics does not predict the urban–rural disparity in use level. This finding is contrary to the result in the knowledge disparity O-B model, where the demographic variables have a big contribution to the overall knowledge disparity. If rural respondents had the same level of focus on food safety as urban residents, the disparity in use of nutrition labels would have reduced by 14.56% (*p*-value < 0.01). If rural respondents were to have the same frequent shopping locations as urban residents, then 23.04% of the disparity in use level would disappear (*p*-value < 0.01). Income is not a significant predictor of the use disparity between the two groups.

The last two columns in Table 4 show the decomposition results for the gaps in perceived benefits of nutrition labels between urban and rural respondents. In the final O-B model, we add both knowledge and use as two independent variables. As observed in Table 4, the overall benefit disparity between urban and rural residents is 0.166. A total of 71.61% of the overall disparity in perceived benefits can be explained by the differences in the explanatory variables included in the model. Knowledge and use are the two biggest contributors, which, together, account for more than 50% of the overall urban–rural disparity in perceived benefits (*p*-value < 0.01). It suggests that the average benefit level would increase by 0.087 if all rural respondents had the same knowledge and use of nutrition labels as urban respondents (*p*-value < 0.01). Further, age explains 21.39% of the disparity in perceived benefits (*p*-value < 0.05). Focus on food safety variables accounts for 13.36% of the overall benefit disparity between two groups (*p*-value < 0.05). The coefficient of the income variable is −0.019 and is significant at the 10% level. The negative coefficient means that closing the income disparity between urban and rural residents would actually increase the disparity in perceived benefits between urban and rural groups, a point that will be discussed in the next section.

## 5. Discussion

This study shows that both urban and rural residents have low levels of knowledge, use and perceived benefits from nutrition labels in China. Specifically, only 15.45% of urban and 14.51% of rural residents generally or totally understand nutrition labels. There are only 36.41% of urban and 27.73% of rural respondents who often or always use nutrition labels. Even smaller percentages of urban and rural residents report benefiting a lot or very much from nutrition labels (urban, 23.11%; rural, 20.69%). The reported knowledge of nutrition labels is lower compared with previous studies that found 19.2% of respondents from Beijing and Baoding (two big cities in Northern China) [22] and 35.3% of parents of primary and secondary school students in Shanghai [23] moderately or totally understand nutrition labels. The lower knowledge of nutrition labels found in our study is likely due to a more geographically diverse sampling that includes both urban and rural areas across China. Liu et al. [22] also found that 28.5% of respondents regularly or always *use* nutrition labels, compared with 36.41% of urban and 27.73% of rural residents in our study. Of note, Liu et al. [22] collected data in 2012, while ours was gathered in 2016. The more frequent use of nutrition labels found in our study may reflect more use of nutrition labels over the years among Chinese consumers.

Despite the progress in the use of nutrition labels over the years, significant disparities persist in the knowledge, use and perceived benefits of nutrition labels between urban and rural consumers. We find that, compared with their urban counterparts, 9.43% *more* of rural respondents do not understand nutrition labels, 14.89% *more* of rural respondents do not use nutrition labels, and 8.87% *more* of rural residents perceive no or slight benefits from nutrition labels. This disparity in nutrition labels knowledge, use and perceived benefits reinforces concerns about urban–rural gaps in diet-related chronic diseases and nutrition [21,37,38,39]. For example, Wang et al. [21] find that after adjusting for age and gender, rural residents have higher prevalence of hypertension, heart disease, cerebrovascular disease, peptic ulcer and chronic cholecystitis among other conditions. Their findings combined with the current study suggest that disparities in the knowledge and use of nutrition labels may contribute to disparities in diet quality and related health problems between urban and rural consumers. Thus, appropriate interventions are needed to address this persistent inequality in knowledge, use and perceived benefits of nutrition labels between urban and rural consumers, and close the gaps in diet quality and related health problems in China.

So, how could the urban–rural disparities in nutrition labels be reduced? The O-B analysis provides important insights into this question. Focus on food safety, demographics, frequent shopping locations and income each contributes to 34.45%, 30.77%, 23.04% and 10.63% of the overall disparity in nutrition label knowledge between urban and rural respondents. The results suggest that improving focus on food safety, enhancing education, increasing availability of large supermarkets and online shopping venues and raising income in rural areas are promising strategies to reduce the disparity in nutrition label knowledge. In fact, the present study shows that, after improving those explanatory variables among rural consumers, 92.84% of the disparity in nutrition label knowledge would disappear. Our results are consistent with previous work that finds education and income are positive predictors of nutrition label knowledge [23,25]. We also offer some new insights to the literature—we discovered that focus on food safety issues and shopping locations are also significant predictors of nutrition label knowledge. Thus, the improvement of those predictors is a fruitful area for future interventions to increase nutrition label knowledge and mitigate the gap between urban and rural consumers.

The present study finds that urban–rural divergence in nutrition label knowledge, frequent shopping locations and focus on food safety significantly contribute to the disparity in nutrition label use. These results support previous studies that find nutrition label knowledge is a significant predictor of nutrition label use [22,27], but also offer new insights that focus on food safety and frequent shopping locations are important factors in helping close the urban–rural gap. If rural respondents had the same shopping locations and focus on food safety as urban counterparts, 32.1% of the disparity in nutrition label use would be gone. Another 30% of the disparity in nutrition label usage would disappear if rural respondents’ nutrition label knowledge were at par with urban consumers. These results collectively suggest that interventions that target at improving nutrition label knowledge, shopping locations and focus on food safety among rural consumers would reduce the urban–rural disparity in nutrition label use.

In terms of the disparity in perceived benefits of nutrition labels, knowledge and use are the two biggest contributors, each accounting for 29.69% and 22.76% of the disparity, respectively. Further, demographics, focus on food safety, and frequent shopping locations jointly explain 36.57% of the disparity. The current study contributes to the literature by finding that if rural consumers’ knowledge and use of nutrition labels, demographics, focus on food safety, and frequent shopping locations were the same as their urban counterparts, 71.61% of the urban–rural disparity in perceived benefits would have disappeared. Thus, policy interventions that address the disparities in knowledge and use of nutrition labels, such as improving awareness of food safety, could also be helpful to mitigate the gap in perceived benefits. Interestingly, the current study shows that increasing rural consumers’ income would actually increase the disparity in perceived benefits from nutrition labels. It is possible that high-income people are willing to spend more money to purchase food with higher perceived quality, such as organic food. In this regard, they are confident with the quality of food and do not use or recognize benefits from nutrition labels [27].

This study has several limitations. The analysis is based on self-reported data, and there could be social desirability bias that respondents overreport the knowledge and use of nutrition labels, among other variables. Moreover, our data were collected in 2016, and the income gaps between urban and rural residents have shrunk over the years since 2016. Therefore the gaps in nutrition labels may also have decreased over time. However, we argue that the important predictors of the gaps found in this study remain important to explain the disparities in the knowledge, use and perceived benefits of nutrition labels. Furthermore, while the model predictors explain a significant part of the urban–rural disparity, there are still some unexplained portions of the gaps. Omitted variables and measurement errors could contribute to the “unexplained part” of the disparity, but O-B model does not offer any further insights into which of these conjectures might be the most plausible. Another limitation of the current approach is that the outcome variables are ordered but are treated as continuous variables because the O-B approach based on ordered logit regression model is unavailable. The current analysis can only infer associations rather than causality between knowledge, use and perceived benefits of nutrition labels and predictors. Additionally, our sample includes individuals who are under 18 years old and future study is warranted to study the heterogeneous behaviors between children and adults. The average respondent in the sample had higher income and education than the national average person. Therefore, future research is needed to study the knowledge, use and perceived benefits of nutrition labels based on a more nationally representative dataset. Finally, several questions in our survey use a Likert scale of five points, which might influence our results because of the higher percentage of responses at the middle point of the scale. Despite the limitations, this study is the first to use an innovative method to analyze the urban–rural disparities in nutrition labels, and utilizes a large-scale survey from 10 provinces across China.

## 6. Conclusions

The current study finds that there are significant disparities in the knowledge, use and perceived benefits of nutrition labels between urban and rural consumers in China. Rural consumers have much lower knowledge, use and perceived benefits of nutrition labels. Reducing the disparities in education and income and raising awareness of food safety issues in rural areas are promising ways to close the urban–rural disparities in nutrition label knowledge and use. Urban–rural divergence in perceived benefits can be further reduced by closing gaps in knowledge and use of nutrition labels. Future research is warranted to assess the optimal set of policies that reduce the urban–rural gaps in the predictors discovered in this study, which contributes to equity in diet quality and health.

## Figures and Tables

**Table 1:** Variable Definitions.

Variables	Description
**Outcome variables**	
Knowledge	Knowledge of nutrition labels (do not understand = 1, understand slightly = 2, understand moderately = 3, generally understand = 4, totally understand = 5)
Use	Use frequency of nutrition labels (never = 1, rarely = 2, sometimes = 3, often = 4, always = 5)
Benefit	Perceived benefits of nutrition labels (no benefits = 1, benefit slightly = 2, benefit moderately = 3, benefit a lot = 4, benefit very much = 5)
**Explanatory variables**	
Demographics	
Rural	Living for more than 6 months (urban = 0, rural = 1)
Age	Age of respondent (years)
BMI	Weight of respondent (Kg)/(height of respondent (Meter))^2^
High school graduates	Education = 1 if the individual at least graduated from high school, 0 otherwise.
Health status	Very poor = 1, poor = 2, fair = 3, good = 4, excellent = 5
Have children	Are there any children at home? (< 18 years old) (yes = 1, no = 0)
Have seniors	Are there any seniors at home? (>= 60 years old) (yes = 1, no = 0)
Focus on food safety	Do you pay attention to food safety issues? (never = 1, rarely = 2, sometimes = 3, often = 4, always = 5)
Frequent shopping locations	Where do you often buy food (each place is a binary variable) (small supermarkets; large supermarkets; online; farmers’ markets; corner stores; street vendors)
Income (in CNY 10,000/USD 1540)	Annual household expenditure
Province indicators	Ten indicator variables

Notes: In China, the farmers’ market is a physical retail marketplace intended to sell foods directly from farmers to consumers. It can be an outdoor or indoor marketplace. Consumers can buy vegetables, fruits, seafood, cooked foods, condiments and so on. The street vendors are several farmers who sell some foods produced by themselves on the roadside. Usually, the number of farmers in the farmers’ market is larger than the street vendors.

**Table 2:** The knowledge, use and perceived benefits of nutrition labels among rural and urban respondents.

	Urban		Rural	
	Observations	Percentage (%)	Observations	Percentage (%)
**Knowledge**				
Do not understand	68	7.24	99	14.22
Understand a little	290	30.88	232	33.33
Understand moderately	436	46.43	264	37.93
Understand a lot	124	13.21	92	13.22
Understand	21	2.24	9	1.29
**Use**				
Never	33	3.51	80	11.49
Rarely	178	18.96	180	25.86
Sometimes	386	41.11	243	34.91
Often	269	28.65	139	19.97
Always	73	7.77	54	7.76
**Benefit**				
No benefits	39	4.15	66	9.48
Benefit slightly	215	22.90	184	26.44
Benefit moderately	468	49.84	302	43.39
Benefit a lot	183	19.49	119	17.10
Benefit very much	34	3.62	25	3.59
Total	939	100.00	696	100.00

**Table 3:** Sample means and regression estimates for the knowledge, use and perceived benefits of nutrition labels among urban and rural respondents.

				Knowledge		Use		Benefit	
Variables	Mean Rural; N = 696	Mean Urban; N = 939	*p*-Value	Coeff. Rural	Coeff. Urban	Coeff. Rural	Coeff. Urban	Coeff. Rural	Coeff. Urban
(1)	(2)	(3)	(4)	(5)	(6)	(7)	(8)	(9)	(10)
Knowledge	2.540	2.723	<0.001 ***			0.606 ***	0.410 **	0.189 ***	0.213 ***
Use	2.867	3.182	<0.001 ***					0.207 ***	0.108 ***
Benefit	2.789	2.955	<0.001 ***						
Age (year)	40.580	31.822	<0.001 ***	−0.001	−0.002	−0.001	−0.000	−0.007 **	−0.003
BMI	23.105	22.311	0.001 ***	−0.003	0.006	−0.002	−0.003	−0.004	−0.005
High school graduates	0.517	0.888	<0.001 ***	0.141	−0.009	−0.080	−0.091	−0.170 **	0.088
Have children	0.565	0.517	0.054 *	0.061	−0.049	0.019	−0.036	0.044	−0.022
Have seniors	0.477	0.347	<0.001 ***	−0.196 ***	0.032	0.036	−0.036	0.034	0.045
Health status									
Very poor	0.019	0.010	0.115						
Poor	0.056	0.024	0.001 ***	−0.201	−0.259	0.055	0.357	−0.202	−0.047
Fair	0.326	0.263	0.005 ***	0.122	−0.465 *	0.247	0.287	−0.123	−0.134
Good	0.421	0.537	<0.001 ***	0.028	−0.312	0.226	0.434	0.039	−0.125
Excellent	0.178	0.166	0.524	0.028	−0.040	0.264	0.505 *	−0.055	−0.198
Focus on food safety									
Never	0.060	0.014	<0.001 ***						
Rarely	0.124	0.088	0.021 **	0.560 ***	0.610 **	0.477 ***	0.271	−0.089	0.038
Sometimes	0.297	0.302	0.826	0.562 ***	0.869 ***	0.409 ***	0.369	0.092	0.269
Often	0.396	0.440	0.031 **	0.750 ***	1.146 ***	0.555 ***	0.680 ***	0.155	0.354
Always	0.132	0.155	0.187	1.066 ***	1.394 ***	0.858 ***	1.006 ***	0.235	**0.444**
Frequent shopping locations									
Small supermarkets	0.489	0.495	0.789	−0.050	−0.093	0.106	0.045	−0.111	0.032
Large supermarkets	0.603	0.869	<0.001 ***	0.158 *	0.032	0.264 ***	−0.053	0.066	−0.022
Online	0.211	0.405	<0.001 ***	0.184 **	0.013	0.039	−0.005	0.085	0.021
Farmers’ markets	0.517	0.479	0.129	−0.027	0.017	0.064	0.100 *	0.109	−0.023
Corner stores	0.445	0.312	<0.001 ***	−0.138 *	0.022	−0.092	−0.196 ***	0.062	−0.024
Street vendors	0.152	0.155	0.860	−0.056	−0.176 **	−0.119	0.025	−0.125	−0.162 **
Income (CNY 10,000)	4.272	7.029	<0.001 ***	−0.011	0.008 *	−0.003	0.009 **	0.002	−0.009 **
Province indicators									
Inner Mongolia	0.105	0.130	0.123						
Sichuan	0.026	0.062	<0.001 ***	−0.292	−0.122	0.393 *	0.011	−0.636 ***	−0.264 *
Shanxi	0.131	0.113	0.273	0.098	−0.034	−0.174	−0.004	−0.224	−0.204 *
Guangdong	0.135	0.132	0.860	−0.276 *	−0.108	−0.019	0.145	−0.368 **	−0.128
Guangxi	0.135	0.103	0.048 **	0.025	−0.014	0.078	0.152	−0.140	−0.027
Jiangsu	0.092	0.094	0.904	0.018	−0.055	0.198	0.034	−0.212	−0.227 **
Hebei	0.080	0.082	0.910	−0.126	−0.110	−0.031	−0.119 *	0.304 *	−0.102
Hunan	0.103	0.089	0.341	0.258 *	−0.129	0.170	0.223 **	−0.059	−0.075
Guizhou	0.086	0.106	0.172	0.024	0.005	0.354 **	0.242	−0.262	−0.312 ***
Heilongjiang	0.106	0.088	0.224	0.097	0.487 ***	−0.137	−0.045	−0.148	0.132

Notes: OLS regression coefficients are presented in columns (5)–(10). *** *p* < 0.01; ** *p* < 0.05; and * *p* < 0.10. The coefficients in bold are the ones significant at least at 10% level.

**Table 4:** Oaxaca–Blinder decomposition results of urban–rural disparities in knowledge, use and benefit level.

Variables	Knowledge		Use		Benefit	
	Coeff.	Percentage (%)	Coeff.	Percentage (%)	Coeff.	Percentage (%)
(1)	(2)	(3)	(4)	(5)	(6)	(7)
Use					0.049 ***	29.689
Knowledge			0.093 ***	29.584	0.038 ***	22.762
Demographics	0.056 ***	30.767	−0.008	−2.445	0.024	14.475
Age	0.012	6.645	0.005	1.481	0.036 **	21.386
BMI	−0.001	−0.574	0.003	0.870	0.004	2.330
High school graduates	0.036	19.728	−0.027	−8.512	−0.017	−10.315
Health status	0.003	1.532	0.011 *	3.516	0.007	4.054
Have children	0.000	−0.067	−0.001	−0.245	0.000	−0.187
Have seniors	0.006	3.503	0.001	0.445	−0.005	−2.793
Focus on food safety	0.063 ***	34.454	0.046 ***	14.560	0.022 **	13.359
Never	0.032 ***	17.267	0.024 ***	7.505	0.007	3.968
Rarely	0.005	2.687	0.002	0.775	0.006 *	3.613
Sometimes	0.000	0.050	0.000	−0.117	0.000	0.103
Often	0.014 **	7.545	0.009 *	2.803	0.005	3.144
Always	0.013	6.905	0.011	3.594	0.004	2.530
Frequent Shopping Locations	0.042 **	23.036	0.055 ***	17.548	0.015	8.734
Small supermarkets	0.000	−0.269	0.000	0.158	0.000	−0.061
Large supermarkets	0.025	13.455	0.033 **	10.345	0.011	6.675
Online	0.015	8.211	0.003	0.880	0.009	5.432
Farmers’ markets	0.000	0.036	−0.003	−1.088	−0.001	−0.719
Corner stores	0.003	1.859	0.023 ***	7.267	−0.004	−2.277
Street vendors	0.000	−0.257	0.000	−0.013	−0.001	−0.316
Income (CNY 10,000)	0.019 *	10.629	0.016	5.050	−0.019 *	−11.196
Province indicators	−0.011	−6.042	0.005	1.704	−0.010	−6.211
Explained gap	0.170 ***	**92.843**	0.208 ***	**66.001**	**0.119**	**71.613**
Unexplained gap	**0.013**	**7.157**	0.107 **	**33.999**	**0.047**	**28.387**
Total gap	**0.183**	**100.00**	**0.315**	**100.00**	**0.166**	**100.00**

Notes: *** *p* < 0.01; ** *p* < 0.05; and * *p* < 0.10. The coefficients in bold are the ones that are significant at least at 10% level.

## Data Availability

The data presented in this study are available on request from the corresponding author.

## References

[1] Zhang J., Zhai L., Osewe M., Liu A. (2020). Analysis of factors influencing food nutritional labels use in Nanjing, China. Foods.

[2] Langenbrunner J.C., Marquez P.V., Wang S. (2011). Toward a Healthy and Harmonious Life in China: Stemming the Rising Tide of Non-Communicable Diseases.

[3] The State Council of the People’s Republic of China (2020). Report on Nutrition and Chronic Diseases of Chinese Residents (2020). http://www.gov.cn/xinwen/2020-12/24/content_5572983.htm.

[4] Afshin A., Sur P.J., Fay K.A., Cornaby L., Ferrara G., Salama J.S., Mullany E.C., Abate K.H., Abbafati C., Abebe Z. (2019). Health effects of dietary risks in 195 countries, 1990–2017: A systematic analysis for the Global Burden of Disease Study 2017. Lancet.

[5] He M., Su D., Zou Y., Huang L., Zhao D., Wang W., Fang Y., Huang E., Gu W., Han D. (2019). Comparative study on burden of diet-related chronic diseases in China between 1990 and 2016. Wei Sheng Yan Jiu J. Hyg..

[6] Kim S.Y., Nayga R.M., Capps O. (2000). The effect of food label use on nutrient intakes: An endogenous switching regression analysis. J. Agric. Resour. Econ..

[7] Pérez-Escamilla R., Haldeman L. (2002). Food label use modifies association of income with dietary quality. J. Nutr..

[8] Cooke R., Papadaki A. (2014). Nutrition label use mediates the positive relationship between nutrition knowledge and attitudes towards healthy eating with dietary quality among university students in the UK. Appetite.

[9] Neuhouser M.L., Kristal A.R., Patterson R.E. (1999). Use of food nutrition labels is associated with lower fat intake. Am. Diet. Assoc..

[10] Graham D.J., Laska M.N. (2012). Nutrition label use partially mediates the relationship between attitude toward healthy eating and overall dietary quality among college students. J. Acad. Nutr. Diet..

[11] Kang H.T., Shim J.Y., Lee Y.J., Linton J.A., Park B.J., Lee H.R. (2013). Reading nutrition labels is associated with a lower risk of metabolic syndrome in Korean adults: The 2007–2008 Korean NHANES. Nutr. Metab. Cardiovasc. Dis..

[12] Wahlich C., Gardner B., McGowan L. (2013). How, when and why do young women use nutrition information on food labels? A qualitative analysis. Psychol. Health.

[13] Cecchini M., Warin L. (2016). Impact of food labelling systems on food choices and eating behaviours: A systematic review and meta-analysis of randomized studies. Obes. Rev..

[14] (2016). Implementation Guidance and Example Analysis of National Standards for Food Safety and Nutrition Standard for Pre-Packaged Food.

[15] Chen X.L., Peng Y., Bai P.Q. (2013). Effect analysis of nutritional intervention on urbanized farmers in Pudong New Area. Mod. Prev. Med..

[16] Fan Y.H., Qin M., Xu X.U., Wang Z.G. (2015). Principal component analysis on difference of urban and rural residents’ concern about food nutrition labels. Food Nutr. China.

[17] Kanbur R., Zhang X. (1999). Which regional inequality? The evolution of rural–urban and inland–coastal inequality in China from 1983 to 1995. J. Comp. Econ..

[18] Center for Health Statistics and Information (2009). An Analysis Report of National Health Services Survey in China.

[19] Huang J., Rozelle S. (2010). Agricultural development, nutrition, and the policies behind China’s success. Asian J. Agric. Dev..

[20] Zhao Y. (1999). Leaving the countryside: Rural-to-urban migration decisions in China. Am. Econ. Rev..

[21] Wang S., Kou C., Liu Y., Li B., Tao Y., D’Arcy C., Shi J., Wu Y., Liu J., Zhu Y. (2015). Rural–urban differences in the prevalence of chronic disease in northeast China. Pac. J. Public Health.

[22] Liu R., Hoefkens C., Verbeke W. (2015). Chinese consumers’ understanding and use of a food nutrition label and their determinants. Food Qual. Prefer..

[23] Ma J., Zhu Z., Chen X., Guo Y., Zhang H., Zhang Y., Zang J. (2018). A cross-sectional survey of nutrition labelling use and its associated factors on parents of school students in Shanghai, China. Public Health Nutr..

[24] Lin C.T., Lee J.Y., Yen S.T. (2004). Do dietary intakes affect search for nutrient information on food labels?. Soc. Sci. Med..

[25] Gracia A., Loureiro M., Nayga R.M. (2007). Do consumers perceive benefits from the implementation of a EU mandatory nutritional labelling program?. Food Policy.

[26] Vemula S.R., Gavaravarapu S.M., Mendu V.V., Mathur P., Avula L. (2014). Use of food label information by urban consumers in India—A study among supermarket shoppers. Public Health Nutr..

[27] Drichoutis A.C., Nayga R.M., Lazaridis P. (2010). Can nutritional label use influence body weight outcomes?. Kyklos.

[28] Satia J.A., Galanko J.A., Neuhouser M.L. (2005). Food nutrition label use is associated with demographic, behavioral, and psychosocial factors and dietary intake among African Americans in North Carolina. J. Am. Diet. Assoc..

[29] Fortin N., Lemieux T., Firpo S. (2011). Decomposition methods in economics. Handbook of Labor Economics.

[30] Sen B. (2014). Using the Oaxaca–Blinder decomposition as an empirical tool to analyze racial disparities in obesity. Obesity.

[31] Jiang W.J., Luh Y.H. (2017). Gender digital divide in a patriarchal society: What can we learn from Blinder–Oaxaca decomposition?. Qual. Quant..

[32] Blinder A.S. (1973). Wage discrimination: Reduced form and structural estimates. J. Hum. Resour..

[33] Oaxaca R. (1973). Male-female wage differentials in urban labor markets. Int. Econ. Rev..

[34] Neumark D. (1988). Employers’ discriminatory behavior and the estimation of wage discrimination. J. Hum. Resour..

[35] Lewis H.G. (1986). Union relative wage effects. Handbook of Labor Economics.

[36] Fryer R.G., Levitt S.D. (2004). Understanding the black-white test score gap in the first two years of school. Rev. Econ. Stat..

[37] Hou X. (2008). Urban—Rural disparity of overweight, hypertension, undiagnosed hypertension, and untreated hypertension in China. Asia Pac. J. Public Health.

[38] Liu H., Fang H., Zhao Z. (2013). Urban–rural disparities of child health and nutritional status in China from 1989 to 2006. Econ. Hum. Biol..

[39] Zhang Y.X., Wang Z.X., Zhao J.S., Chu Z.H. (2016). Prevalence of overweight and obesity among children and adolescents in Shandong, China: Urban–rural disparity. J. Trop. Pediatr..

